# Probing CO_2_ Reduction Pathways for Copper Catalysis Using an Ionic Liquid as a Chemical Trapping Agent

**DOI:** 10.1002/anie.202009498

**Published:** 2020-09-03

**Authors:** Gui‐Rong Zhang, Sascha‐Dominic Straub, Liu‐Liu Shen, Yannick Hermans, Patrick Schmatz, Andreas M. Reichert, Jan P. Hofmann, Ioannis Katsounaros, Bastian J. M. Etzold

**Affiliations:** ^1^ Ernst-Berl-Institut für Technische und Makromolekulare Chemie Technical University of Darmstadt Alarich-Weiss-Str. 8 64287 Darmstadt Germany; ^2^ Surface Science Laboratory Department of Materials and Earth Sciences Technical University of Darmstadt Otto-Berndt-Str. 3 64287 Darmstadt Germany; ^3^ Helmholtz-Institute Erlangen-Nürnberg for Renewable Energy (IEK-11) Forschungszentrum Jülich GmbH Egerlandstraße 3 91058 Erlangen Germany

**Keywords:** CO_2_ reduction, copper, foams, ionic liquids, reaction mechanisms

## Abstract

The key to fully leveraging the potential of the electrochemical CO_2_ reduction reaction (CO2RR) to achieve a sustainable solar‐power‐based economy is the development of high‐performance electrocatalysts. The development process relies heavily on trial and error methods due to poor mechanistic understanding of the reaction. Demonstrated here is that ionic liquids (ILs) can be employed as a chemical trapping agent to probe CO2RR mechanistic pathways. This method is implemented by introducing a small amount of an IL ([BMIm][NTf_2_]) to a copper foam catalyst, on which a wide range of CO2RR products, including formate, CO, alcohols, and hydrocarbons, can be produced. The IL can selectively suppress the formation of ethylene, ethanol and n‐propanol while having little impact on others. Thus, reaction networks leading to various products can be disentangled. The results shed new light on the mechanistic understanding of the CO2RR, and provide guidelines for modulating the CO2RR properties. Chemical trapping using an IL adds to the toolbox to deduce the mechanistic understanding of electrocatalysis and could be applied to other reactions as well.

## Graphical abstract

The presence of a small amount of ionic liquid significantly alters the product spectrum of CO_2_ reduction over a Cu catalyst. The ionic liquid acts as a chemical trapping agent, selectively suppressing the formation of C_2+_ products that involve carbene as a key intermediate. The response in product distribution to ionic liquid modification offers a new way to disentangle the complex reaction network of CO_2_ reduction by Cu catalysts.

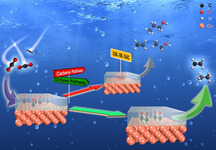

## Introduction

The electrochemical CO_2_ reduction reaction (CO2RR) provides a promising solution to offset the increased atmospheric CO_2_ concentration, and also represents an excellent option for storing intermittent renewable electricity (e.g. solar, wind energy) by producing value‐added chemicals.^1^ However, poor energy conversion efficiency and broad product spectrum are major barriers to achieving economic viability of the CO2RR. Intensive effort has been spent searching for high performance electrocatalysts.^2^ Copper (Cu) is identified as the only metal that produces hydrocarbons and alcohols with appreciable Faradaic efficiency (FE),^3^ due to its moderate binding strength with key intermediate species.^4^ Despite its unique catalytic properties, mechanistic understanding of the reaction pathways which provides the basis of steering the CO2RR toward desired products, remains controversial. Although the adsorbed *CO species is well‐accepted as a key intermediate leading to various C_2+_ products, it remains an open challenge to elucidate the mechanistic pathways from *CO to C_2+_ products on Cu. Especially, the formation mechanisms of ethylene and ethanol have long been the subject under debate in both experimental and theoretical studies.^5^

Mechanistic understanding of the CO2RR are derived almost exclusively through in situ/operando spectroscopic techniques (e.g., IR, Raman).^6^ Early in situ spectroscopic studies of Cu electrodes suggest that hydrogenation of *CO to *CH_2_ would be the precursor to ethylene and ethanol,6c, 7 while others suggest that formation of these C_2_ species would mainly proceed through forming a *CO dimer (*C_2_O_2_^−^) which is subsequently protonated to *CO‐COH.^8^ These discrepancies may stem from the inherent limitations of spectroscopic techniques. The limitations include the interference from the solvent or spectator species,8b, 9 limited temporal/spatial resolution due to the low coverage and short residual time of key intermediates,6c, 6d and ill‐defined background signals that are sensitive to electrode pretreatment history and cell configurations,6d, 10 and all these may add to the uncertainty of the measurement and make interpretation of resultant spectra a non‐trivial task.6d Complementary ways of analyzing the CO2RR mechanism are highly desirable.

Chemical trapping is regarded as an effective way to study reaction mechanisms. It originated in organic chemistry and was widely applied in catalysis.^11^ The reaction mechanism is deduced using a compound (trapping agent) that reacts specifically with one or more reaction intermediate(s) to form a stable product(s). The trapping agent stops/decelerates specific reactions, and reaction mechanisms can then be deduced by examining the products. Bell et al. demonstrated in their exemplary works that the production of hydrocarbons from CO hydrogenation involved adsorbed methylene species as a key intermediate, as shown by the suppressed formation of hydrocarbons in presence of methylene scavengers.^12^ This chemical trapping method has not yet been applied to electrocatalysis, largely due to the lack of suitable chemical trapping agents that can selectively interact with specific intermediates without being oxidized/reduced under electrochemical conditions. Inspired by previous works where ionic liquids (ILs) were employed as surface modifiers to modulate the catalytic properties of a variety of electrocatalysts, an IL is used here as a chemical trapping agent to analyze the CO2RR pathways in Cu catalysts. This idea is realized by analyzing the IL‐induced perturbation in the product spectrum. The rationales for choosing ILs also include their coordination ability with CO2RR intermediates and good stability over a wide potential window.^13^ ILs have been used as either pure electrolyte or electrolyte additive to change the CO2RR properties in various metal catalysts (e.g., Ag, Pb).^14^ ILs are reported to lower the overpotential and explicitly favor the formation of CO, presumably through coordinating with reduction intermediates (e.g., CO_2_^−.^) by either stabilizing the intermediates or preventing their spatial approach.13c, 15

In the current study, the IL is introduced by immobilizing a small amount of 1‐butyl‐3‐methylimidazolium bis(trifluoromethylsulfonyl)imide ([BMIm][NTf_2_]) on a Cu‐Foam catalyst (see Figure S1 in the Supporting Information). This method follows the concept of “solid catalyst with ionic liquid layer (SCILL)”, which was first invented in heterogeneous catalysis,^16^ and was soon successfully transferred to electrocatalysis particularly in improving electrocatalysts for the oxygen reduction reaction (ORR).^17^ The hydrophobic nature of the IL and capillary force ensure the confinement of the IL within the catalysts even in aqueous electrolytes.17d, 17e We demonstrate that IL can act as a chemical trapping agent in the CO2RR. Its presence significantly alters the product spectrum by selectively suppressing the formation of ethylene, ethanol, and n‐propanol, without disturbing either FE or partial current density of the others. These findings demonstrate selective interactions between the IL and one or more reaction intermediate(s), while the altered product distribution provides a unique perspective to track the CO2RR pathways. This work may represent a simple approach to gaining mechanistic insights into the CO2RR, and also paves a new way in modulating the CO2RR activity and selectivity.

## Results and Discussion

**Figure 1:**
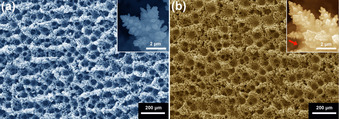
Representative SEM images of a) Cu‐Foam and b) Cu‐Foam‐IL; the insets show magnified images, and the red arrow in the inset of panel (b) marks a curved meniscus of the IL.

Cu foams were chosen because of the unique catalytic property of Cu and the abundance of porous structure which is beneficial to IL immobilization. Cu‐Foams were prepared using a hydrogen evolution reaction (HER) assisted electrodeposition method,^18^ with a Cu plate as the substrate and copper sulfate as the precursor (Figure S2). [BMIm][NTf_2_] was chosen because of its hydrophobic nature and ability to coordinate with CO_2_ and/or its anion radical.^15^, ^19^ Figure 1 a displays the representative scanning electron microscopy (SEM) image of pristine Cu‐Foam, featuring an open porous structure. A magnified image (the inset in Figure 1 a) discloses a dendritic structure composing of irregularly shaped particles. Meanwhile, IL modification has not induced any pronounced difference in either the morphology or their average macropore sizes (31.8±8.1 μm vs. 31.7±8.4 μm; Figures 1 b and S3). The IL can be seen (the inset in Figure 1 b), existing as blur on the dendritic nanostructures. Characteristic elements of the IL (F, N, and S) can be identified on Cu‐Foam‐IL using both energy dispersive X‐ray spectroscopic (EDS) and X‐ray photoelectron spectroscopy (XPS) (Figures S4 & S5), confirming the successful incorporation of the IL in Cu‐Foam. To explore the spatial distribution of the IL, EDS elemental mapping analyses were performed (Figure S6). The EDS signals of F and S from the [BMIm][NTf_2_], are distributed over the porous Cu foams and surround the macropores without any localized aggregation, suggesting a homogeneous distribution of the IL. To probe any possible change in the surface electronic structure of Cu after IL modification, XPS (Cu 2p_3/2_) and Auger spectra (Cu LMM) of Cu were recorded (Figure S7). Both samples exhibit a major XPS peak at a binding energy (BE) of 932.5 eV, which associates with Cu^0^/Cu^+^. The Cu LMM Auger spectra confirm that the surface Cu on both samples mainly exists as Cu^+^ (i.e. Cu_2_O),^20^ which is not surprising since the oxidation of Cu to Cu_2_O occurs immediately upon air exposure.^21^ A minor shoulder peak at a BE of 934.7 eV, which associates with Cu(OH)_2_,^20^ can also be observed on pristine Cu‐Foam, indicating that a small portion of Cu_2_O in Cu‐Foam are prone to further oxidation to form Cu(OH)_2_. This consequent oxidation process was also reported by Tannenbaum et al. when studying the initial oxidation behavior of Cu in air.^21^ Intriguingly, this shoulder peak is absent on Cu‐Foam‐IL, implying that the IL can help suppress surface oxidation, which is in line with our previous study on Pt‐based catalysts.17e, 17f, 17g Notwithstanding this difference, considering the well documented readiness of copper oxide reduction under CO2RR conditions,^10^, ^22^ the presence of a small portion of Cu(OH)_2_ species on initial Cu‐Foam is not expected to play a significant role in altering the product distribution. The CO2RR performance on Cu is sensitive to surface facets of Cu.^23^ To find out whether the IL can change the Cu surface by selectively blocking certain facets, we performed Pb_UPD_ stripping experiments on both samples (Figure S8). The comparable integrated areas of Pb_UPD_ stripping peaks verify that (selective) blocking of Cu facets by the IL can be ruled out.

**Figure 2:**
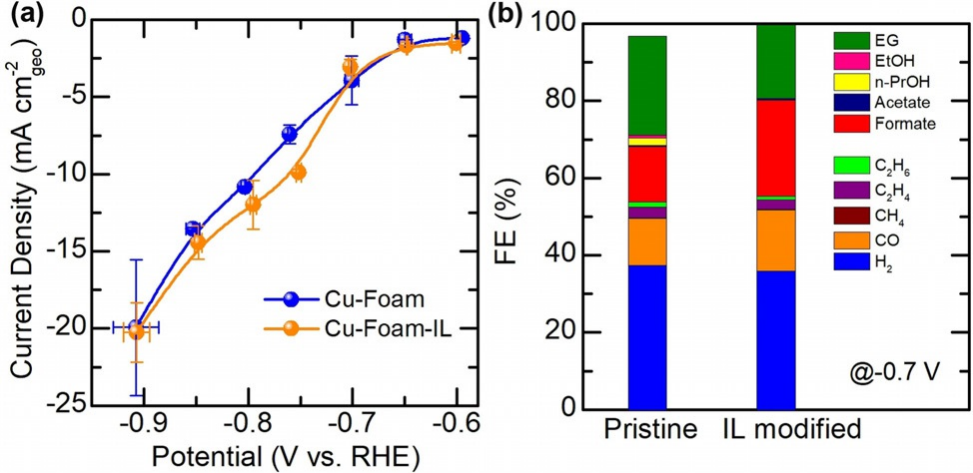
a) Current densities recorded at various electrolysis potentials, and b) Faradaic efficiency of CO2RR over Cu‐Foam and Cu‐Foam‐IL at −0.7 V. Electrolysis was performed for 1 hour in 0.1 m CO_2_ saturated KHCO_3_ solution.

The CO_2_ electrolysis experiments were performed in a gas‐tight electrochemical cell with anode and cathode separated by an anion exchange membrane (Figure S9). Figure 2 a compares the overall current densities of both samples obtained from chronoamperometry experiments at various potentials (Figure S10). Despite the fluctuation, the electrolysis current densities are more or less comparable at the beginning and end of the electrolysis on both samples. This result indicates that Cu foams are stable during the electrolysis regardless of IL modification, which is also evidenced by the intact dendritic structures of both Cu foams after the electrolysis (Figure S11). The stability of the IL on Cu foams during the CO2RR was also probed by performing post‐reaction analyses of Cu‐Foam‐IL using both XPS and diffuse reflectance infrared Fourier transform spectroscopy techniques. The characteristic signals of IL can be clearly resolved using both techniques after the electrolysis (Figure S12), implying that the IL can be well‐maintained within the Cu foams during electrolysis. The overall current densities are comparable between these two samples, despite a slight current increase in Cu‐Foam‐IL at potentials of −0.7 and −0.8 V versus reversible hydrogen electrode (vs. RHE). These results verify that the presence of the IL has not induced any change in mass transport properties of reactant molecules (CO_2_) from bulk electrolyte to Cu‐Foam surfaces, and also imply that the majority of the CO_2_ molecules may approach the catalyst surface in a free form instead of an IL‐coordinated form. Figure 2 b compares the FEs of various products on both samples at −0.7 V. A variety of products, including CO, formate, ethylene glycol (EG), ethylene, ethane, ethanol, n‐propanol, methane, and acetate can be detected, with CO, formate, and EG identified as the major products (in addition to hydrogen). Various CO2RR products can usually be observed in Cu foams, while the major product depends on their morphology, active surface area, and foam thickness.2b, 18, 24 Intriguingly, herein we observe that EG, which is usually identified as a minor product in Cu catalysts, is produced with impressively high FEs (≈20 %) on both samples. These results showcase that Cu foams are a versatile platform in producing value‐added CO2RR products.

**Figure 3:**
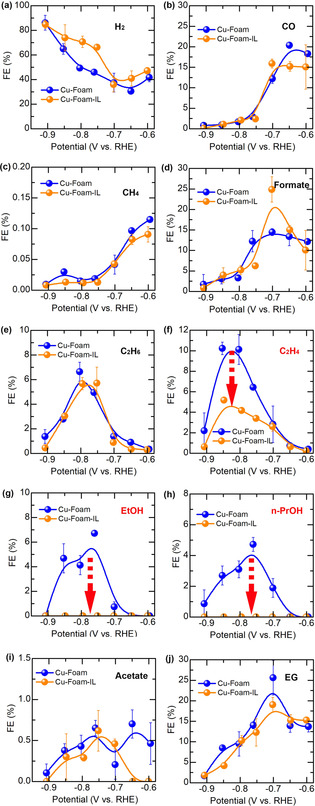
Summary of the FEs for the CO2RR products on both Cu‐Foam catalysts at different potentials. The suppressed products are marked in red. The arrows emphasize the changes in FEs of the suppressed products.

The potential dependent FEs of various products on pristine and IL‐modified Cu‐Foams are compared in Figure 3. The HER, a major competing reaction of the CO2RR, still dominates the product spectra on both catalysts. A surge in H_2_ production is observed at electrode potentials lower than −0.7 V, relating to the liberation of surface sites from adsorbed *CO.^25^ Meanwhile, the HER is promoted by IL modification. This may stem from the inherent acidity and superior proton transfer capability of the IL being used, which offers greater proton availability for the HER.14a, 26 The H_2_ production rates on both samples converge at lower electrode potentials (< −0.85 V), indicating that at higher reaction rate, the HER is mainly limited by the diffusion of proton (or proton source) from bulk solution to the catalyst surface, and the influence of IL modification is not pronounced. Similar potential‐dependent FEs for major CO2RR products, including formate, EG, CO, ethylene, and ethane, can be observed on both samples despite some minor difference in FEs for EG and formate at around −0.7 V, due to the liberation of strongly adsorbed *CO intermediate from Cu surfaces. Different from other studies of the CO2RR on Cu catalysts, on which methane is a major product, in the current work, methane is produced with a rather low FE (<1 %) on both catalysts. Similarly, Broekmann et al. also observed that C_1_ pathway to methane was almost completely suppressed on Cu foams.^18^ The morphology or surface faceting of Cu catalysts plays a crucial role in determining the product selectivity of CO2RR.^27^ For instance, Cu(100) facets favor the formation of ethylene while Cu(111) facets facilitate the formation of methane.27b This structure sensitive behavior of the CO2RR on Cu catalysts originates from the differences in binding energy of *CO and/or energetic barrier for the C−C coupling or hydrogenation step between different Cu facets.8b, 28 Herein, the low FEs of methane on both catalysts imply that the Cu foams after the initial reduction of surface Cu_2_O species during the CO2RR might be enclosed by abundant Cu(100) facets as suggested by Broekmann et al.^18^, ^24^ The comparable FEs and onset potentials for major products such as CO and formate on Cu‐Foam and Cu‐Foam‐IL also verify that the presence of IL has not induced any fundamental structural change on the Cu foam itself, and at the same time, the possible blockage or surface rearrangement of specific faceting by IL molecules during the CO2RR can be excluded. The most striking effect induced by IL modification is that ethanol and n‐propanol, giving a maximum FE of 7 % and 5 % on pristine Cu‐Foam, respectively, are completely absent on Cu‐Foam‐IL (Figures 3 h and i). Meanwhile, the FE of ethylene is solely suppressed in the high overpotential region (< −0.7 V), with the highest FE decreasing from 10.2 % to 5.2 % after IL modification (Figure 3 d), while little difference can be observed in the low overpotential region (i.e. from −0.6 to −0.7 V). The same conclusion can also be drawn by comparing the partial current densities of CO2RR products (Figure S13). The IL has selectively slowed down the production rate of ethylene in the high overpotential region and ceased the production of ethanol and n‐propanol. These results demonstrate that the feasibility of the IL as a chemical trapping agent, which provides the basis for deducing the CO2RR pathways by analyzing the altered product spectrum in presence of the IL.

**Figure 4:**
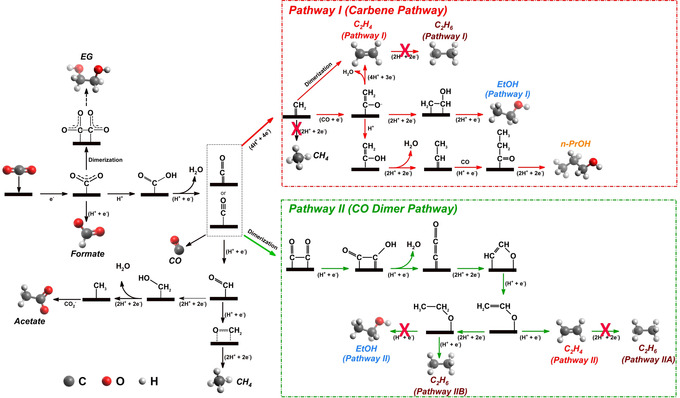
Proposed reaction roadmaps of CO2RR on Cu catalysts. Selected intermediates are presented for clarity. Unfeasible pathways are marked by red crosses.

Despite understandings of reaction pathways on Cu catalysts are rife with controversy, some consensus has been reached, which enables discussion of the observed chemical trapping results. Transferring the first electron to CO_2_ to form CO_2_^−.^ anion is considered as the rate‐determining step for CO_2_ activation because of the high reorganization energy needed to activate a linear CO_2_ molecule to form CO_2_^−.^ anion with bent coordination geometry.8d, 15, 29 Moreover, CO is identified as a key intermediate during the reduction of CO_2_ to various C_2+_ products, since CO is the only C_1_ molecule that gives similar product spectrum as CO_2_ on a Cu catalyst.3a, 3d However, it remains elusive how the adsorbed CO intermediate is further converted into various products. Intrigued by the altered product spectrum after IL modification, we clarify several elusive reduction pathways by referring to the widely reported yet controversial mechanism in literature, as summarized in Figure 4.

Among various products, ethylene shows the most interesting response to IL modification. Its formation is only suppressed at high overpotentials, while at low overpotentials both FE (Figure 3 d) and the partial current density of ethylene (Figure S13d) are almost the same regardless of IL modification. This result strongly suggests that ethylene could form by two separate pathways at high and low overpotentials. A dual pathway mechanism for ethylene production was proposed by Koper et al. when studying CO reduction on Cu.28b One pathway (Pathway II) involves the dimerization of two adjacent CO at low overpotentials, which is later reduced and protonated to form ethylene. The dimerization would proceed by forming a hydrogenated CO dimer (*CO‐COH) as confirmed by spectroscopic and theoretical studies.8b On the other pathway (Pathway I), CO is converted into either *CHO4a, 28b or *COH^30^ at high overpotentials, which is then reduced to carbene‐like *CH_2_ species, followed by either C−C coupling between two *CH_2_, or CO insertion as in the Fischer–Tropsch process, to produce ethylene.^30^ The dual pathway mechanism may also hold its validity for ethylene production in Cu‐Foams. The IL could selectively quench one or more intermediates in Pathway I, which eventually suppresses the formation of ethylene at high overpotentials, while it appears that Pathway II, which starts at relatively low overpotentials and involves the C−C coupling through CO dimerization, is undisturbed by the IL.

Ethane is not a typical CO2RR product on Cu catalysts.^31^ The production of ethane with a significant FE is explicitly observed on nanostructured porous Cu catalysts.^31^, ^32^ Ethane can be seen as a reduction product of ethylene after two more protonation steps. The porous structure of Cu catalysts seems to increase the retention time of pre‐formed products in a confined space. Therefore, for a long time, the formation of ethane has been attributed to the re‐adsorption and reduction of pre‐formed ethylene on Cu catalysts (Pathways I and IIA).^31^, ^32^ However, both FE and partial current density of ethane are actually insensitive to IL modification (Figures 3 e and S13e). The entirely different responses of FEs for ethylene and ethane to IL modification imply that ethane is formed via an independent pathway. Recent works report that ethane is produced by the CO dimerization pathway involving ethoxy intermediate,^33^ which reconciles with our observation that the pathway involving CO dimerization is undisturbed by the IL. These findings suggest that production of ethane would mainly proceed through Pathway IIB (Figure 4).

Ethanol is considered to share the similar formation mechanism as ethylene.3d, 8d Two reaction pathways, which involve either formation of carbene intermediate (*CH_2_) (Pathway I) or dimerization of two adjacent CO (Pathway II), are usually proposed (Figure 4). We found that formation of ethanol is completely suppressed on Cu‐Foam‐IL, which suggests that IL traps the key intermediate(s) leading to the formation of ethanol. Similarly, n‐propanol is not produced on Cu‐Foam‐IL. It is generally accepted that the formation of n‐propanol undergoes intramolecular C−C coupling between CO and hydrogenated carbon (e.g., carbene *CH_2_), followed by proton/electron transfer to form propionaldehyde, an intermediate that is further reduced to n‐propanol (Figure 4).3d, 8d, 34 It can be seen that the formation of both ethanol and n‐propanol involves carbene species (*CH_2_), which is also the intermediate to produce ethylene at high overpotentials. The IL‐induced suppression of ethanol, n‐propanol, and ethylene (at high overpotentials, Pathway I) infers that these products likely share one or more common intermediate(s) selectively trapped by the IL.

Regarding the other CO2RR products including CO and formate, their differences in FEs and partial current densities are quite minor or within measurement error between two catalysts, determining their pathways conclusively becomes challenging. Nevertheless, some inspiring information can be deduced. For instance, the formation of CO and formate is insensitive to IL modification, indicating that starting from the adsorption of CO_2_ on Cu surfaces to the formation of adsorbed CO, the IL seems to play a negligible role, or in other words, the IL does not take effect through coordinating with CO_2_ molecules which are more likely transported to the catalysts surface in a free form. Moreover, EG is usually detected as a minor product of the CO2RR.^31^, ^35^ However, herein both Cu foam catalysts exhibit fairly high FE of EG: up to 25 % and 19 % on Cu‐Foam and Cu‐Foam‐IL, respectively. The formation of EG is double‐checked by analyzing the liquid products using GC‐MS (Figure S14). Consensus on the reaction pathway to EG has not yet been reached, although it is inferred that EG formation might proceed through a CO dimerization mechanism.^31^, ^35^ Herein, EG formation is always accompanied by formate, and their FEs exhibit similar potential‐dependent behavior, that is, higher FEs obtained at lower overpotentials and maximum FEs obtained at around −0.7 V. These results imply that these two products probably share the same intermediate, for example, *CO_2_^−^, which has been experimentally confirmed as a key intermediate to produce formate.^36^ Brennecke et al. suggested that C−C coupling could also take place between two adsorbed CO_2_^−^ to form oxalate species.19a The hypothesis here is that EG is produced via dimerization of two adsorbed *CO_2_^−^ species, instead of *CO, followed by multistep reduction and protonation to give EG (Figure 4). The predominant product at −0.7 V switches from EG on Cu‐Foam to formate on Cu‐Foam‐IL. The IL may inhibit the dimerization process of the co‐adsorbed CO_2_^−.^ species by preventing their close approach.19a It is also intriguing to observe that IL modification exhibits little impact on the methane formation. Two reaction pathways are usually proposed for the methane formation. One pathway involves carbene (*CH_2_) as an intermediate, which is further reduced to *CH_3_ and finally to CH_4_. The other pathway is through hydrogenation of *CO to form *CHO, followed by a multiple electron‐proton transfer process to produce CH_4_ (Figure S15). Considering that Pathway I (carbene pathway) has been significantly suppressed by the IL, herein, comparable FEs of methane on both Cu foams leads us to hypothesize that methane is mainly produced through the latter pathway (Figure 4).

**Figure 5:**
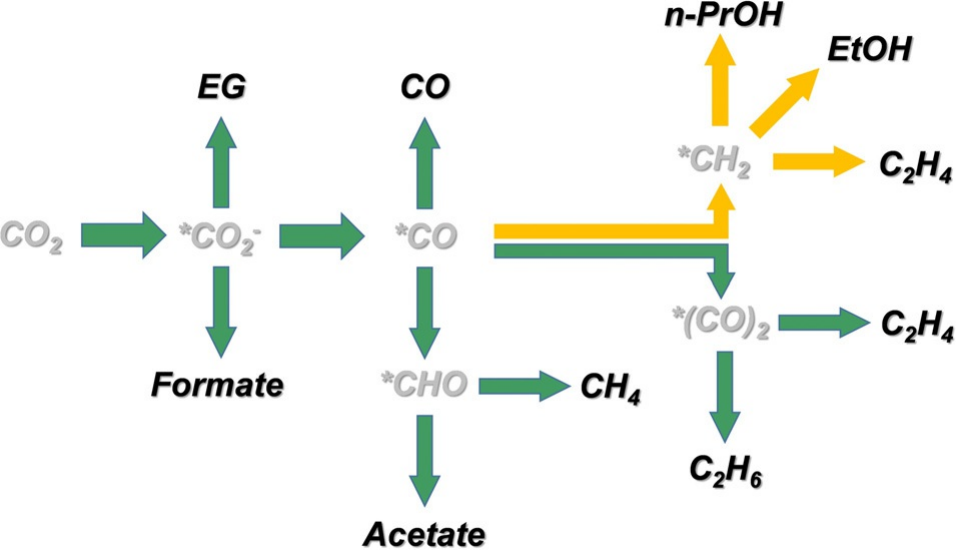
A simplified diagram summarizing the proposed CO2RR pathways on Cu. The IL suppressed pathways and products are highlighted in yellow.

Analyzing the IL‐induced change in CO2RR product distribution provides a unique perspective to gain some unprecedented mechanistic insights into the Cu catalyzed CO2RR which actually bypasses the necessity of explicit understandings about the chemical identity of surface intermediate(s). Based on the above results, a simplified overview of the reaction pathways that lead to varied CO2RR products is summarized in Figure 5, where the IL suppressed products and pathways are highlighted in yellow. It is intriguing to observe that the bifurcation of intermediates leading to the suppressed products starts right after the formation of adsorbed carbene (i.e. *CH_2_) (Figure 4). This hints that the key intermediate(s) are either *CH_2_ or other species (e.g., *COH, *CHO, *C, *CH) that can further be converted to *CH_2_ (Figure S16). Another key question is how the IL molecules can trap the surface intermediate(s). IL molecules are reported to adopt a charge‐separated layered structure with alternating cation‐/anion‐rich layer at electrified surfaces.17f, 37 Accordingly, [BMIm]^+^ cations should be enriched at the innermost (Stern) layer of the electrode‐electrolyte interface when the electrode is negatively polarized (i.e. the CO_2_ electrolysis conditions). Therefore, understanding of how [BMIm]^+^ cations can possibly interact with other species would be crucial to extrapolate the role of ILs during the CO2RR. It is well documented that an imidazolium cation can easily be deprotonated at its C2‐site, thus converting the C2‐site into a reactive center due to its nucleophilicity.^38^ Accordingly, to clarify whether [BMIm]^+^ interacts with surface intermediate(s) via its C2‐site, an imidazolium‐based IL on which the C2‐site at the imidazolium cation ring is “neutralized” by a methyl group (denoted as [BMMIm]^+^, Figure S17a), was used for modifying Cu foams. It turns out that the chemical trapping effect of the IL is not pronounced. Both ethanol and propanol can be detected, and the formation of ethylene at high overpotentials is not suppressed (Figure S18). Furthermore, another IL, [HMIm][NTf_2_] which shares structural similarity with [BMIm][NTf_2_] but features a longer cationic chain, was also tested. Although both ethanol and propanol can still be detected, their FEs are much lower than those on unmodified counterpart, and ethylene formation is also suppressed (Figure S18). Two more common ILs (i.e. [MTBD][NTf_2_], [P_1444_][NTf_2_]) were also tested for comparison. Not surprisingly, no pronounced chemical trapping effect can be identified using either IL (Figure S18). Their product spectra are comparable to that of the unmodified Cu‐Foam, except for a slightly higher FEs of H_2_ on Cu‐Foam modified with [MTBD][NTf_2_], probably due to the protonic nature of this IL. These results lead us to hypothesize that the IL traps the surface key intermediates through bonding with carbene (or other hydrogenated carbon species) on Cu surfaces. This process may involve deprotonation and following alkylation reactions at the C2‐site of the imidazolium ring.^39^

## Conclusion

This work outlines a new strategy to probe CO2RR pathways. The IL alters the product spectrum during the CO2RR on Cu foams. Analyzing the responses of CO2RR products to IL modification is a unique way to gain new insights into CO2RR pathways: 1) Ethanol and n‐propanol form explicitly through a “carbene” mechanism, while formation of ethylene could proceed through two independent pathways which involve carbene and dimerized CO as key intermediates at high and low overpotentials, respectively; 2) The presence of the IL can selectively suppress the formation of those products involving carbene intermediates, likely by forming stable imidazolium‐carbene compound(s); 3) Ethane, which has long been considered a reduction product of re‐adsorbed ethylene during CO2RR, is identified as proceeding with an independent pathway that involves CO dimerization process. Considering the great structural flexibility in ILs, identification of reaction pathways for CO_2_ products by carefully designing task‐specific ILs to selectively interact with intermediate species may be feasible. The success of this will bring IL modification closer to being a generic strategy for analyzing complicated CO_2_ reduction pathways. This approach is transferable to other electrocatalytic reactions and materials. This work demonstrates the possibility of moderating the CO2RR product spectrum by rationally leveraging the IL modification effect, which can be key to finely tuning the catalytic properties of a CO_2_ reduction catalyst at a molecular level.

## Conflict of interest

The authors declare no conflict of interest.

## Supporting information

As a service to our authors and readers, this journal provides supporting information supplied by the authors. Such materials are peer reviewed and may be re‐organized for online delivery, but are not copy‐edited or typeset. Technical support issues arising from supporting information (other than missing files) should be addressed to the authors.

Supplementary
