# Supplementary material for: Probing CO2 Reduction Pathways for Copper Catalysis Using an Ionic Liquid as a Chemical Trapping Agent
Source: Angew Chem Int Ed Engl. 2020 Sep 3;59(41):18095–102. doi: 10.1002/anie.202009498 (PMC7589334; doi:10.1002/anie.202009498)
Supplement: Supplementary file 1 — Supplementary [file ANIE-59-18095-s001.pdf]

Angewandte Chemie
Eine Zeitschrift der Gesellschaft Deutscher Chemiker

Supporting Information

Probing CO_2 Reduction Pathways for Copper Catalysis Using an Ionic Liquid as a Chemical Trapping Agent

*Gui-Rong Zhang[+], Sascha-Dominic Straub[+], Liu-Liu Shen[+], Yannick Hermans, Patrick Schmatz, Andreas M. Reichert, Jan P. Hofmann, Ioannis Katsounaros, and Bastian J. M. Etzold**

anie_202009498_sm_miscellaneous_information.pdf

Supporting Information

1. Synthesis of Cu Foam

Copper plates (99.99 %, Alfa Aesar) were pretreated using a literature protocol[1]. First, the copper plates were cleaned of impurities by ultrasonication in concentrated sulfuric acid (96 %, Suprapur, Merck). The clean copper plates were then anodized in ortho-phosphoric acid (85 %, EMSURE ACS, Merck) by applying a voltage of 3 V vs. Ag/AgCl (3M NaCl) for 3 minutes. Copper foam was synthesized using a hydrogen evolution assisted electrodeposition method[2]. As shown in Figure S1, two copper plates (99.99 %, Alfa Aesar) were fixed in a polypropylene holder at a distance of about 2 cm from each other and immersed in a plating bath containing 1.5 M sulfuric acid (96 %, Suprapur, Merck) and a 0.2 M copper sulfate solution (98 %, VWR). The projected geometric surface area (A_{geo}) was 5.7 cm^2. For the electrodeposition, an HMP 4040 power supply (Rhode & Schwarz) was used and a potential of 12 V was applied between the copper cathode and the copper anode. The electrodeposition time was controlled by LabView software. After electrodeposition, the resulting copper foam was thoroughly rinsed with deionized water (VWR Chemicals) and isopropanol (99.9 %, Carl Roth). The area density of electrodeposited Cu foam is determined to be 2.4 ± 0.1 mg cm^{-2}.

2. Syntheses of ionic liquids

[BMIm][NTf$_2$]. The synthesis was carried out in a round-bottom flask fitted with a reflux condenser for 24 h. Under vigorous stirring, 1-bromobutane (17.1 g, 0.125 mol) was added dropwise over 1 h to a solution of 1-methylimidazole (10.3 g, 0.125 mol) in acetonitrile (40 mL) while keeping the temperature below 5 °C. Afterwards, the temperature was gradually increased up to 50 °C for 5 h. The reaction was completed after 18 h at 70 °C. [BMIm]Br was then obtained by evaporating the solvent in a rotary evaporator (Büchi Rotavapor R-220) at 60 °C for 24 h.

The anion exchange was performed by slowly adding the Li[NTf$_2$] aqueous solution (0.037 mol) to [BMIm]Br (0.037 mol), also dissolved in water. The IL was separated as a viscous fluid phase beneath the water phase. It was washed several times with ultrapure water until the $AgNO_3$ test on the aqueous layer was negative. The resulting IL was dried according the aforementioned procedure for [BMIm]Br followed by high vacuum at room temperature for 24 h.

[HMIm][NTf$_2$]. This synthesis followed the same procedure as for [BMIm][NTf$_2$] but employing bromohexane instead of bromobutane. In this sense, equimolar amounts of the precursors were also used (1-methylimidazole: 10.3 g, 0.125 mol; 1-bromohexane: 20.6 g, 0.125 mol) for the preparation of [HMIm]Br. The anion exchange was performed by mixing the obtained [HMIm]Br (0.032 mol) with the Li[NTf$_2$] aqueous solution (0.032 mol).

[MTBD][NTf$_2$]. The [MTBD][NTf$_2$] IL was synthesized following a literature procedure.[3] Briefly, equimolar amounts of the precursors 7-methyl-1,5,7-triazabicyclo[4.4.0] dec-5-ene [MTBD] (5.2 g, 33.9 mmol) and the lithium salt of bis(trifluoromethylsulfonyl)imide [NTf$_2$] (9.7 g, 33.9 mmol) dissolved in water with HNO_3 were cooled in ice near to 0 °C. HNO_3 was added dropwise to the [MTBD] solution

until neutral pH was reached. Then, the Li[NTf$_2$] solution was slowly added and the IL precipitated as a viscous fluid phase beneath the water phase. The IL was washed several times with ultrapure water and then placed in a rotary evaporator (Büchi Rotavapor R-220) at 60 °C for 24 h to remove residual water.

3. Preparation of IL-modified Cu Foam

The IL-modified Cu Foam was synthesized by coating the Cu Foam sample with ILs. Before IL modification, the total pore volume of the Cu Foam sample was estimated by soaking the Cu Foam with isopropanol: the volume of isopropanol required to fully saturate the Cu Foam was taken as the total pore volume (V_0). An isopropanol solution containing a ceratin amount of the IL was then drop casted on the Cu Foam substrate to prepare the IL-modified Cu Foam. The IL impregnated Cu Foam was air dried for 30 minutes before use. The loading of IL on Cu-Foam is 0.75 mg, and the calculated weight ratio of Cu foam to IL is 1: 0.32.

4. Electrochemical measurements

A design of electrochemical cell similar to that in Jaramillo et. al was used in this work[4]. Polyetherether ketone (PEEK) was used to construct the cell. As illustrated in Figure S9, the cell has a parallel arrangement and 2 cm between the working and counter electrodes, which were separated by an anion exchange membrane (Selemion AMV, AGC inc.). The reference electrode used was a Ag/AgCl (3M NaCl, ALS Co., Ltd) and was placed close to the working electrode in the cathode compartment. The catholyte volume was 4 mL and the copper foam working electrode had an exposed geometric surface area of 1 cm^2. Graphite foil (99.8 %, Alfa Aesar) was used as the counter electrode, which was placed in the anodic compartment, with an anolyte volume of 8 mL. A CO_2 flow of 30 mL/min was established for the catholyte compartment with a mass flow controller (EL-FLOW, Bronkhorst) to ensure sufficient CO_2 transport to the surface of the working electrode. A pure $KHCO_3$ electrolyte (0.1 M, pH = 6.8) was prepared by purging a solution of K_2CO_3 (99.997 %, Alfa Aesar) with CO_2 for up to 30 min.

Electrochemical studies were carried out with a PMC1000 multichannel potentiostat (AMETEK). At the beginning, the ohmic resistance (R_u) was determined by electrochemical impedance spectroscopy (EIS). The R_u value was taken at 10 kHz, as referred in the literature[1,3]. Before electrolysis experiments, copper foam catalysts were pretreated using the cyclic voltammetry (CV) technique in the potential range of -1.8 to -0.5 V vs. Ag/AgCl. The CO_2 electrolysis experiments were carried out for 1 hour using the chronoamperometry (CA) technique. During the electrolysis measurements, 85 % of the ohmic resistance was automatically compensated for by the potentiostat. The remaining uncompensated resistance (R_u, 15 %) was post-corrected according to the following equation:

$$E_{Ag/AgCl,measured} = E_{Ag/AgCl,applied} - R_u \underline{I}_{measured}$$

where $\underline{I}_{measured}$ is the average current throughout the electrolysis experiment. The corrected potential was converted to RHE by

$E_{RHE} = E_{Ag/AgCl,measured} + 0.209 + 0.059pH.$

The stripping experiments of underpotential deposition (UPD) of Pb on Cu foams were performed in an aqueous electrolyte of 0.01 M $HClO_4$ + 1 mM $PbCl_2$. The electrolyte was purged with high purity nitrogen for 15 min before the stripping experiments. For comparison, the capacitance current corresponding to the double layer charging process was determined by recording voltammetry curves in an electrolyte of 0.01 M $HClO_4$ + 2 mM KCl. The voltammetric curves were recorded at a scan rate of 10 mV s^{-1}.

5. CO2RR product analysis

Gas phase products were analyzed using online gas chromatography (Nexis GC-2030, Shimadzu) with a flame ionization detector (FID) and a thermal conductivity detector (TCD). The TCD was used to quantify H_2 and CO, while the FID was used to analyze other gaseous carbonaceous products. Calibration was performed using standard gases with three different concentrations. The gas products were sampled every 13 minutes during the constant potential electrolysis. The liquid products were analyzed using ^{1}H-NMR with a water suppression method. Faradaic efficiencies were calculated based on the partial current of each product, using the following equation:

$$FE\ [\%] = \frac{I_i}{I_{tot}} = \frac{x_i[\%] \cdot z \cdot F \cdot \dot{n}_{CO_2}}{I_{tot}}$$

where I_i is the partial current of a product (i); I_{tot} is the total reduction current; x_i is the molar fraction of certain product; z is the electron transfer number; F is the Faraday constant; $\dot{n}_{CO_2}$ is the molar flow rate of CO_2.

6. Structural analysis

The morphology and elemental mapping of battery cross-section and electrodes were probed using a Scanning Electron Microscopy (Philips XL30 FEG) equipped with an EDAX X-ray detector (CDU Leap XL-30) operated at an accelerating voltage of 30 kV. X-ray photoelectron spectroscopy (XPS) measurements were carried out using a SPECS Phoibos 150 analyzer and a SPECS Focus 500 X-ray source using the monochromatized AlKα line at 1486.7 eV. The Fermi edge of a sputter cleaned silver sample was used to calibrate the binding energies of all photoelectron spectra. Pass energies of 25 eV and 10 eV were used for the survey and core level spectra, respectively. Spectra were evaluated using CasaXPS software. The samples (fresh IL-modified and spent-IL modified copper foam) were investigated by diffuse reflectance infrared Fourier transform spectroscopy (DRIFTS) in a spectral range from 600 cm^{-1} to 4000 cm^{-1} using a Bruker Vertex 70 XSA spectrometer with an MCT detector. Pristine copper foam was used as reference. The resolution was 1.0 cm^{-1}.

7. Supplementary figures

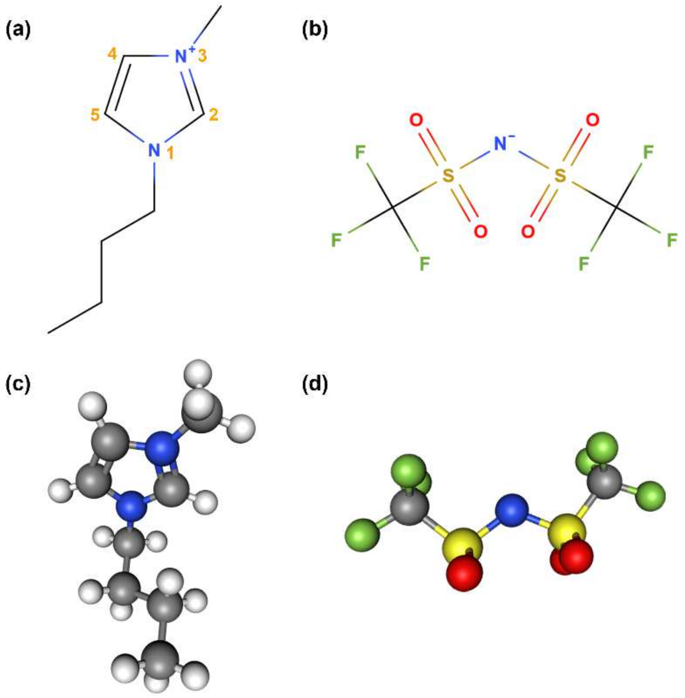

Figure S1. Structural formula of a) [BMIm] cation; b) [NTf_2] anion; and their 3D structure models (c,d).

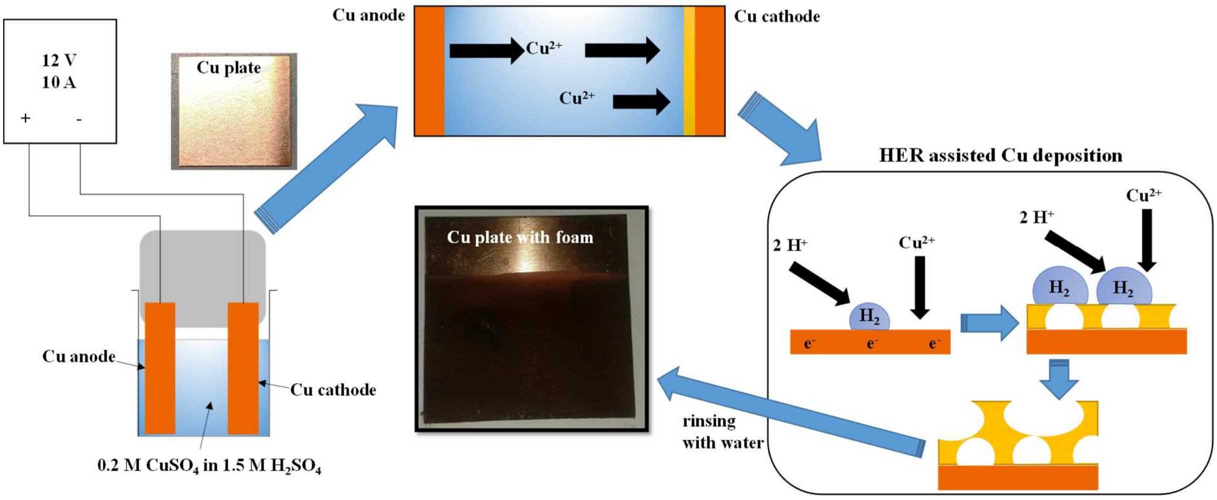

Figure S2. Schematic illustration of synthesis of Cu Foam materials using the hydrogen evolution assisted electrodeposition method.

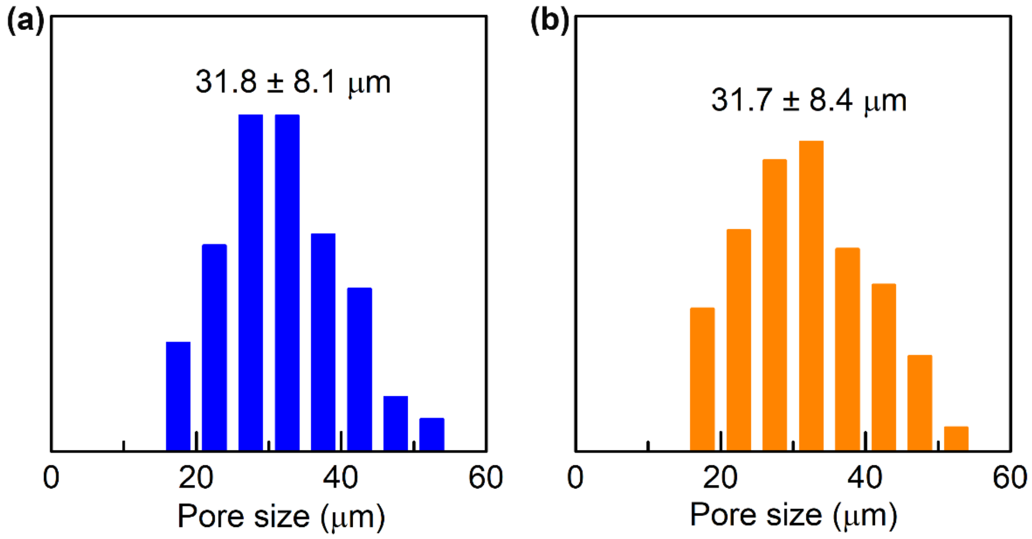

Figure S3. Pore size histograms of a) Cu-Foam and b) Cu-Foam-IL. Over 200 pores randomly taken in different SEM images were measured for pore size distribution analysis for each sample.

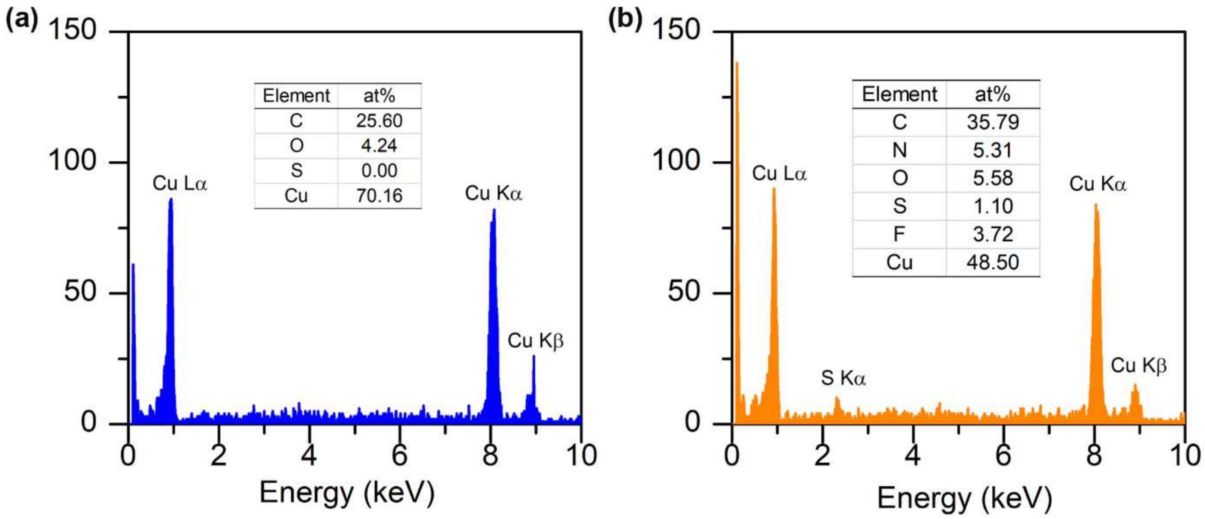

Figure S4. EDS of a) Cu-Foam and b) Cu-Foam-IL. The insets show the elemental analysis data.

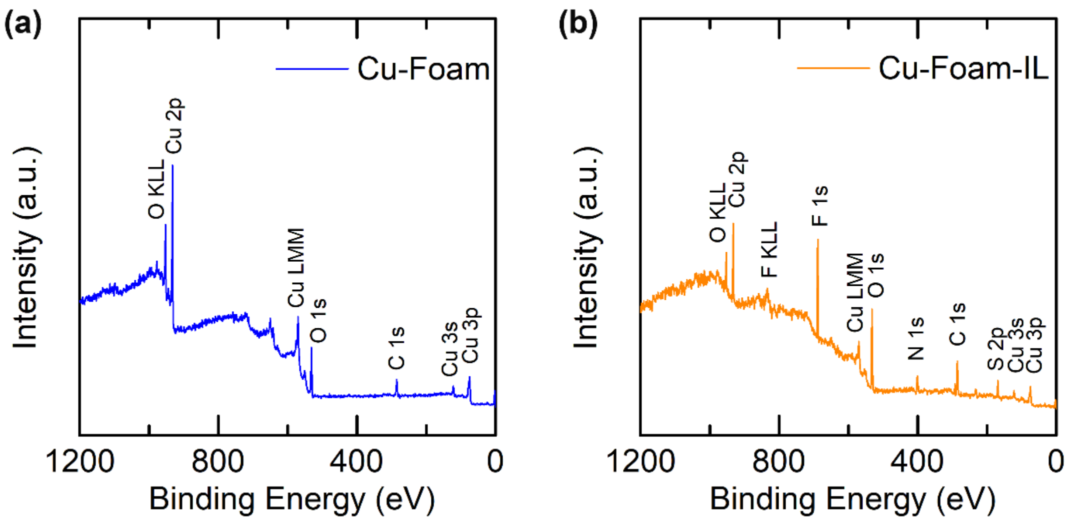

Figure S5. XPS survey spectra of a) Cu-Foam and b) Cu-Foam-IL.

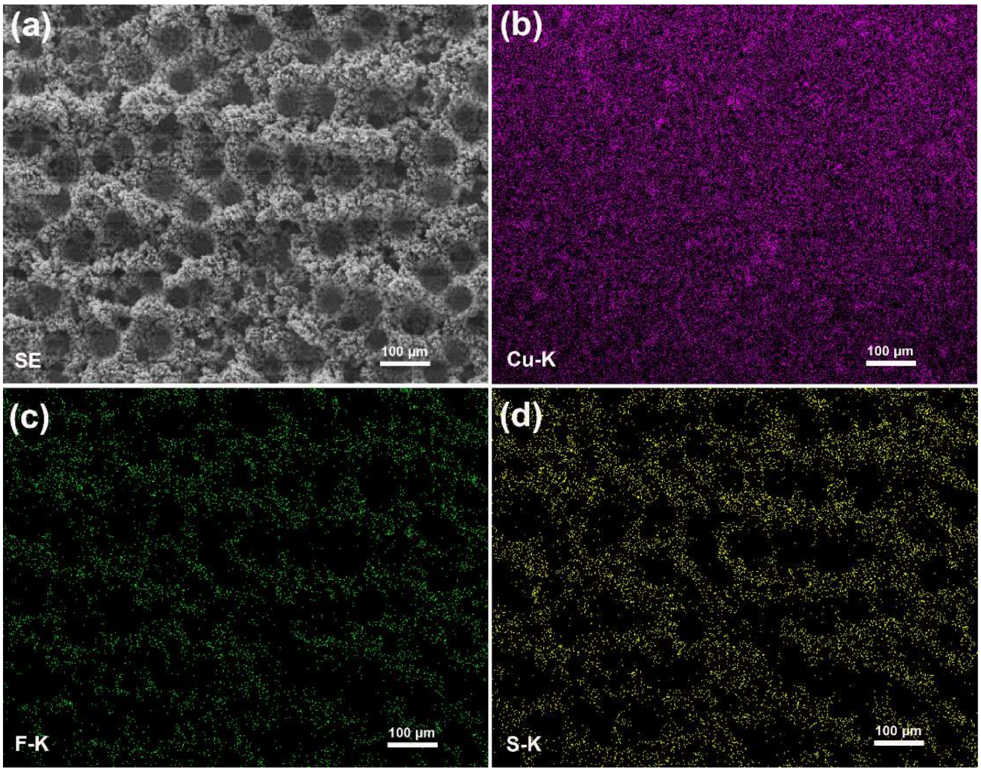

Figure S6. a) SEM and b-d) EDS elemental mapping images of Cu-Foam-IL showing the EDS signals of b) Cu, c) F and d) S.

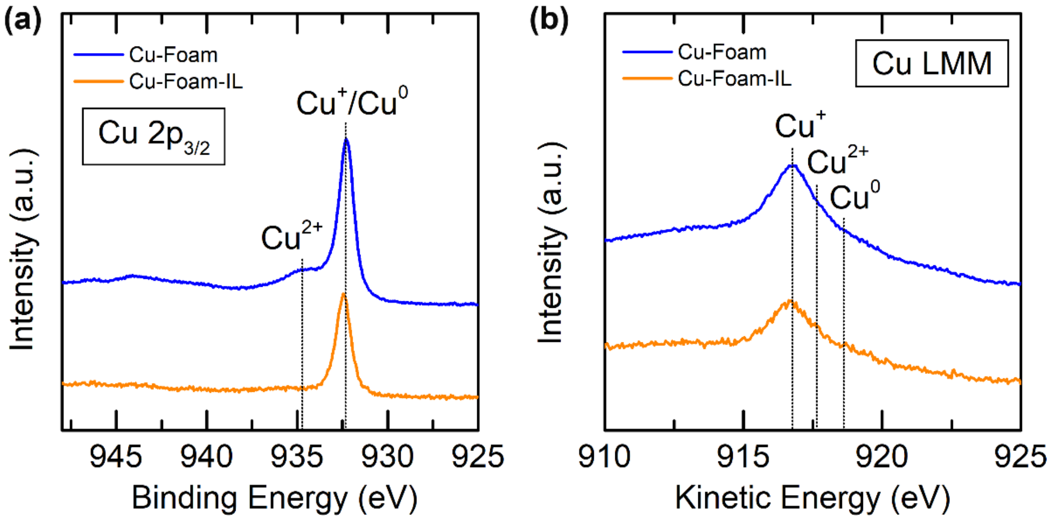

Figure S7. a) XPS and b) Auger spectra of Cu-Foam and Cu-Foam-IL. The vertical lines mark the BE/KE reference values for Cu with different oxidation states.[5]

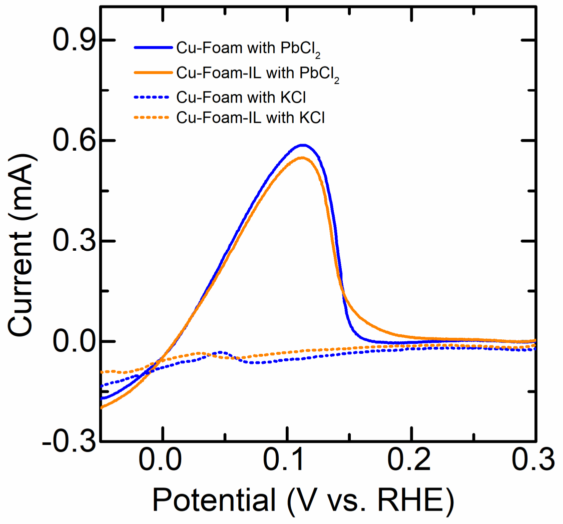

Figure S8. Voltammograms for the stripping of Pb_{UPD} on Cu-Foam and Cu-Foam-IL in 0.01 M $HClO_4$ solution with $PbCl_2$ (solid) or KCl (dotted). Scan rate: 10 mV s^{-1}. Integrated peak areas are determined to be 6.60×10^{-5} and 6.59×10^{-5} V·A for Cu-Foam and Cu-Foam-IL, respectively.

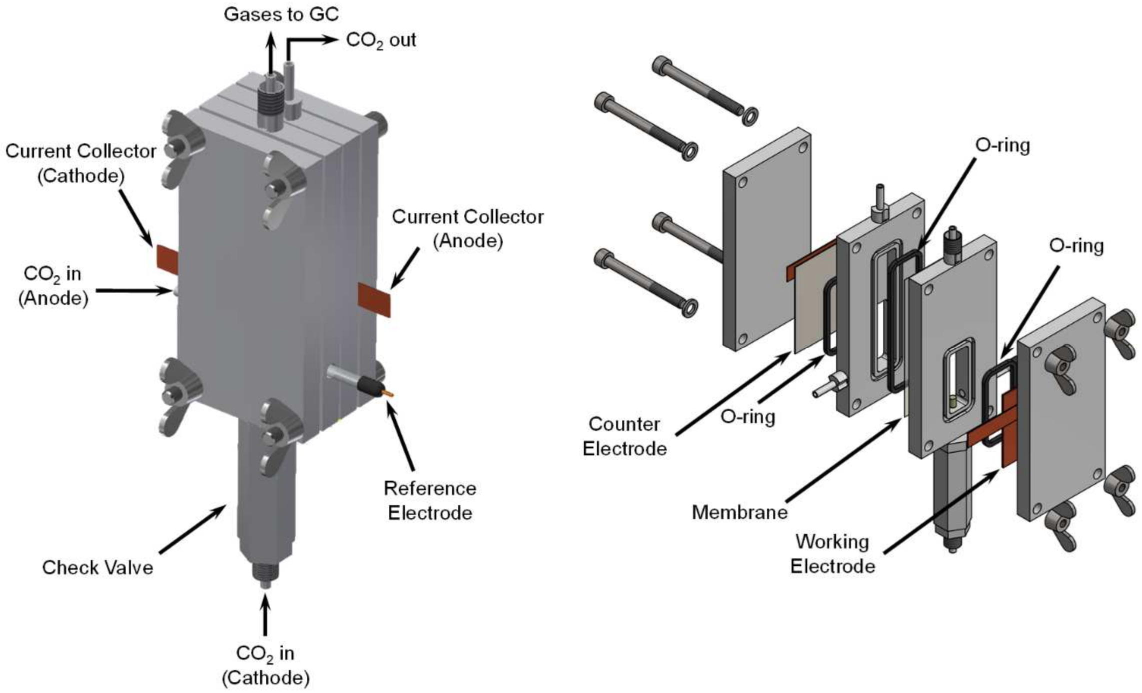

Figure S9. Diagram of the electrochemical cell used for CO_2 electrolysis experiments.

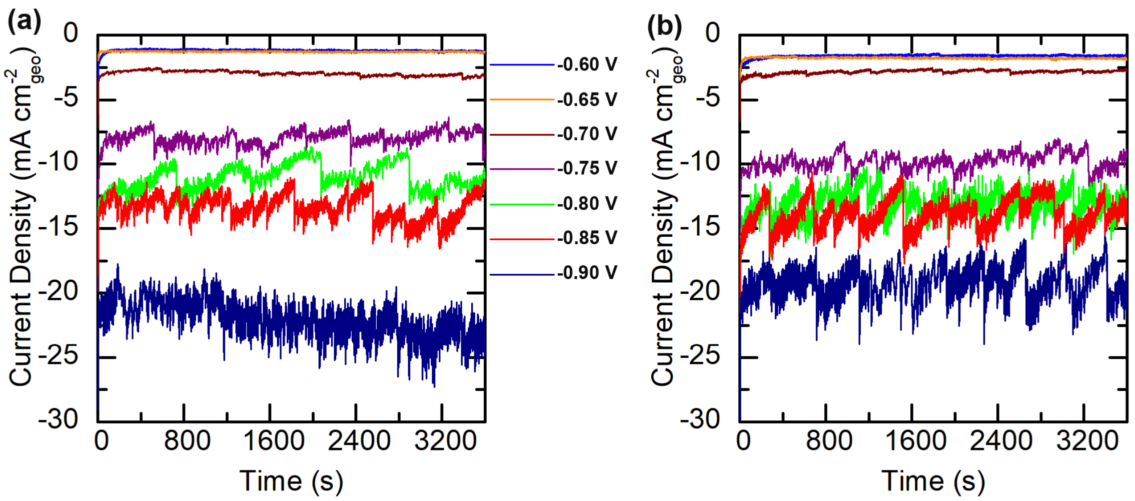

Figure S10. Chronoamperometric (CA) curves for CO_2 electrolysis at different potentials on a) pristine Cu Foam; and b) Cu Foam-IL in CO_2 saturated 0.1 M $KHCO_3$ solution.

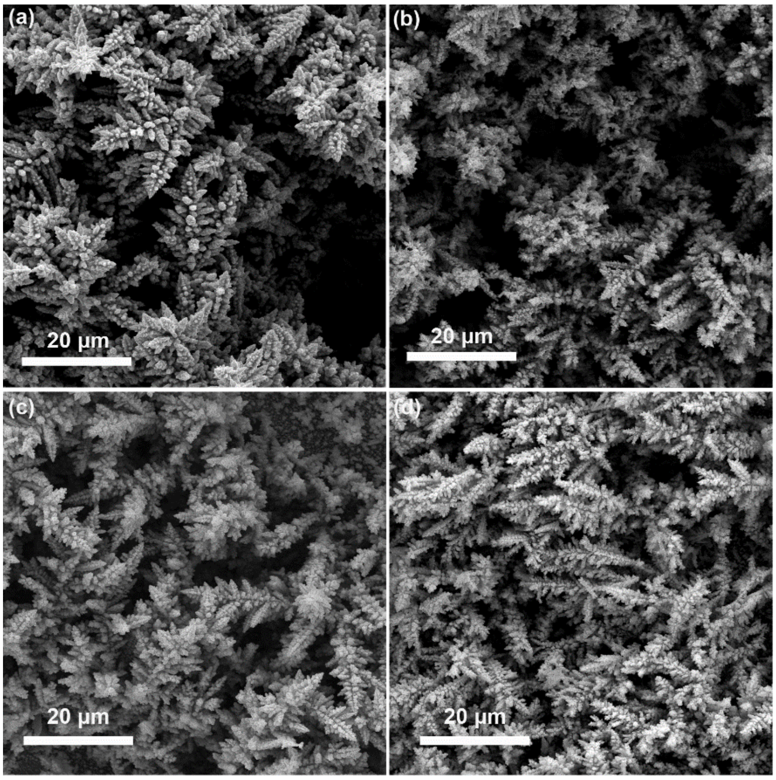

Figure S11. SEM images of a,b)Cu-Foam and c,d) Cu-Foam-IL a,c)before and b,d) after the CO_2 electrolysis.

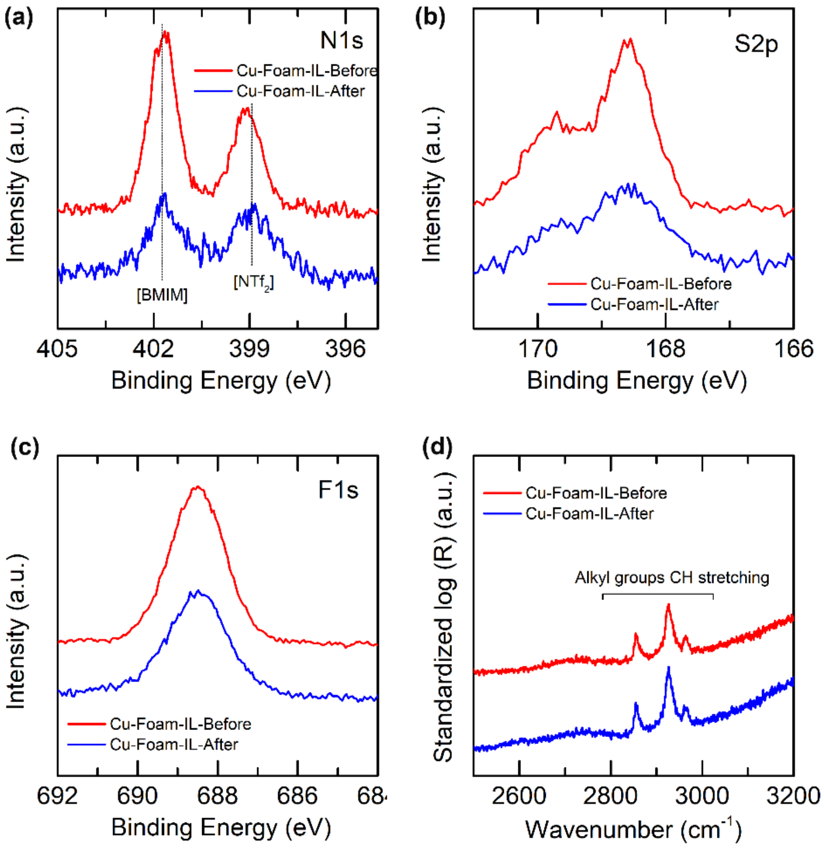

Figure S12. a) N 1s, b) S 2p, c) F 1s XPS and d) DRIFTS of Cu-Foam-IL before and after the CO_2 electrolysis. The vertical lines mark the BE reference values of N 1s for the IL.[6]

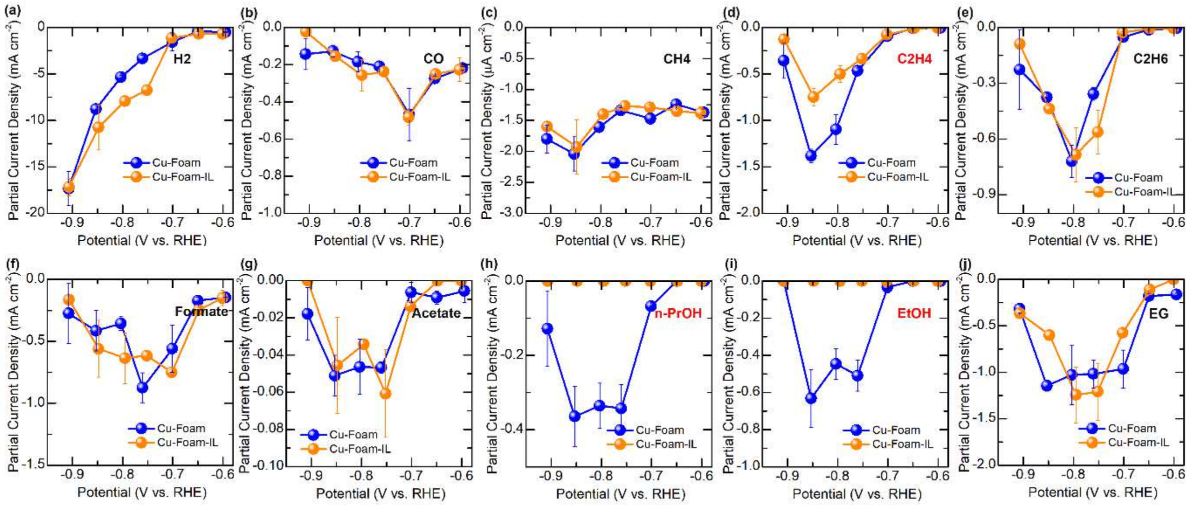

Figure S13. Comparison of partial current densities of a) H_2; b) CO; c) Formate; and d) ethylene glycol on the pristine and IL-modified Cu-Foam.

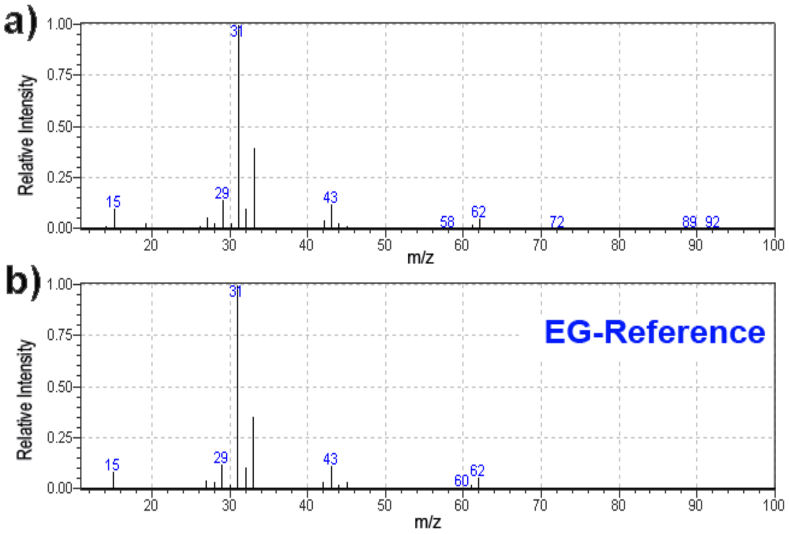

Figure S14. Comparison of MS signals of (a) the CO_2 reduction product; and (b) Ethylene glycol (EG) reference (GCMS Postrun Analysis software database).

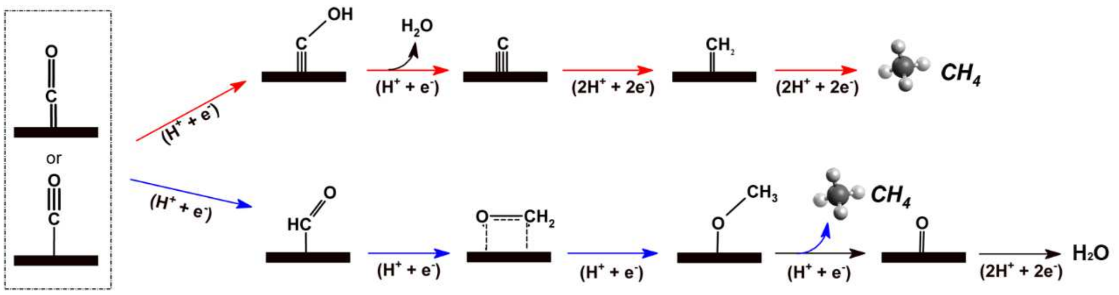

Figure S15. Two possible reaction pathways for the electrocatalytic reduction of CO_2 to methane.

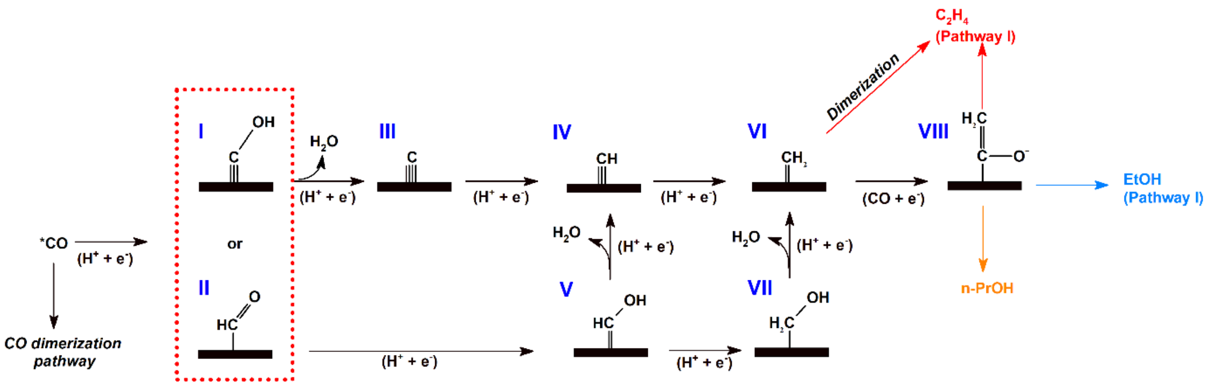

Figure S16. Possible reaction intermediates leading to adsorbed CH_2 and suppressed products.

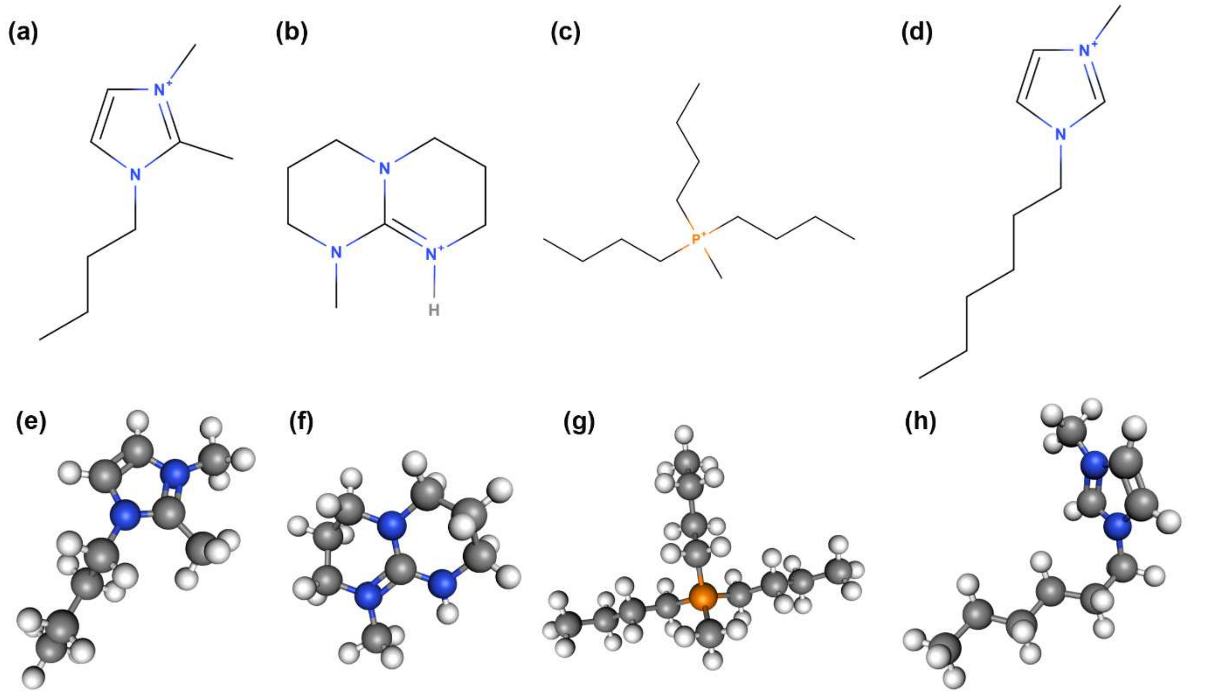

Figure S17. Cationic structures of other ILs being used for IL modification. a) $[BMMIm]^+$, b) $[MTBD]^+$, $[P_{1444}]^+$, and $[HMIM]^+$. e-f) Their corresponding 3D molecular structures.

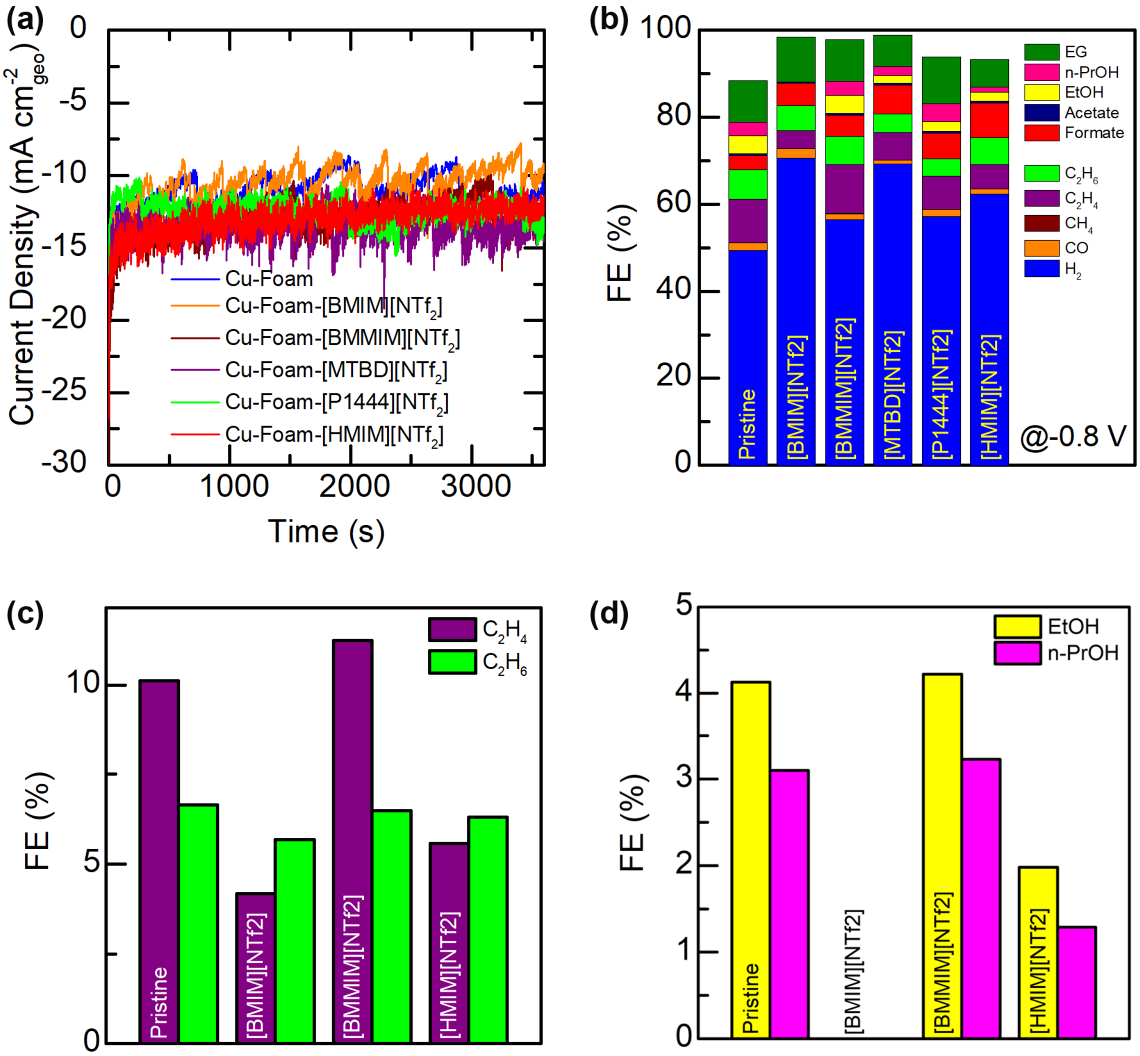

Figure S18. a) CA curves and b) FEs of CO2RR at -0.8 V on pristine and IL modified Cu-Foams. c-d) Comparison of the FEs for C_2H_4, C_2H_6, and alcohols on pristine and IL modified Cu-Foams.

References

[1] K. Jiang, R. B. Sandberg, A. J. Akey, X. Liu, D. C. Bell, J. K. Nørskov, K. Chan, H. Wang, *Nat. Catal.* **2018**, *1*, 111-119.

[2] H.-C. Shin, J. Dong, M. Liu, *Adv. Mater.* **2003**, *15*, 1610-1614.

[3] Y. M. Tan, C. F. Xu, G. X. Chen, N. F. Zheng, Q. J. Xie, *Energy Environ. Sci.* **2012**, *5*, 6923-6927.

[4] K. P. Kuhl, E. R. Cave, D. N. Abram, T. F. Jaramillo, *Energy Environ. Sci.* **2012**, *5*, 7050-7059.

[5] L. Martin, H. Martinez, D. Poinot, B. Pecquenard, F. Le Cras, *J. Phys. Chem. C* **2013**, *117*, 4421-4430.

[6] O. Höfft, S. Bahr, M. Himmerlich, S. Krischok, J. A. Schaefer, V. Kempter, *Langmuir* **2006**, *22*, 7120-7123.
